# Relationship between the Oral and Vaginal Microbiota of South African Adolescents with High Prevalence of Bacterial Vaginosis

**DOI:** 10.3390/microorganisms8071004

**Published:** 2020-07-04

**Authors:** Christina Balle, Rachel Esra, Enock Havyarimana, Shameem Z. Jaumdally, Katie Lennard, Iyaloo N. Konstantinus, Shaun L. Barnabas, Anna-Ursula Happel, Katherine Gill, Tanya Pidwell, Jairam R. Lingappa, Hoyam Gamieldien, Linda-Gail Bekker, Jo-Ann S. Passmore, Heather B. Jaspan

**Affiliations:** 1 Department of Pathology, Institute of Infectious Disease and Molecular Medicine, University of Cape Town, 7925 Cape Town, South Africa; rachel.esra@unige.ch (R.E.); ehavyarimana@gmail.com (E.H.); shameemzjaumdally@gmail.com (S.Z.J.); katieviljoen@gmail.com (K.L.); iyaloombodo@gmail.com (I.N.K.); barnabas.shaun@gmail.com (S.L.B.); anna.happel@uct.ac.za (A.-U.H.); hoyam.gamieldien@uct.ac.za (H.G.); jo-ann.passmore@uct.ac.za (J.-A.S.P.); hbjaspan@gmail.com (H.B.J.); 2 Desmond Tutu HIV Centre, University of Cape Town, 7925 Cape Town, South Africa; Katherine.Gill@hiv-research.org.za (K.G.); Tanya.Pidwell@hiv-research.org.za (T.P.); Linda-Gail.Bekker@hiv-research.org.za (L.-G.B.); 3 Family Clinical Research Center, Stellenbosch University, 7505 Stellenbosch, South Africa; 4 Departments of Pediatrics and Global Health, University of Washington, Seattle, WA 98195, USA; lingappa@uw.edu; 5 National Health Laboratory Service, 7925 Cape Town, South Africa; 6 Seattle Children’s Research Institute, Seattle, WA 98101, USA

**Keywords:** adolescents, microbiota, oral, bacterial vaginosis, periodontitis, South Africa

## Abstract

Bacterial vaginosis (BV) and periodontal disease (PD) are characterised as bacterial dysbioses. Both are associated with an increased risk of poor pregnancy outcomes, yet it is unknown whether PD and BV are related. We characterised the oral microbiota of young South African females with a high prevalence of BV and investigated the association between oral communities and vaginal microbiota. DNA was extracted from vaginal lateral wall, saliva and supragingival plaque samples from 94 adolescent females (aged 15–19 years). 16S rRNA gene sequencing of the V4 hypervariable region was performed for analysis of the oral and vaginal microbiota and BV status was determined by Nugent scoring. The core oral microbiota was predominately comprised of Firmicutes followed by Proteobacteria and Bacteroidetes. The salivary microbiota of participants with BV was more diverse than those with lactobacillus-dominated communities (*p* = 0.030). PD-associated bacterial species, including *Prevotella intermedia* and *Porphyromonas endodontalis* were enriched in the supragingival microbiota of women with non-optimal vaginal communities compared to those with *Lactobacillus*-dominant communities, while *Pseudomonas aeruginosa* and *Prevotella intermedia* were enriched in the saliva of women with non-optimal vaginal microbiota. These data suggest a relationship between oral and vaginal dysbiosis, warranting further investigation into whether they are casually related.

## 1. Introduction

Despite the number of exposures the oral cavity experiences on a daily basis, the core human oral microbiota has been described as consisting of six major phyla, representing 96% of bacteria found in the saliva of healthy individuals, namely—Firmicutes, Bacteroidetes, Proteobacteria, Actinobacteria, Spirochaetes and Fusobacteria [1,2,3,4]. An altered oral microbiota has been identified as a marker of several diseases, including diabetes [5], cancer [6], HIV [7], autoimmune disease [8] and systemic inflammation [9]. Furthermore, distinct oral microbiotas have been observed in non-disease states, for example in pregnant and lactating women [10], indicating that homeostatic alterations may manifest in the oral microbiota. Periodontal disease (PD) is a polymicrobial condition in which development of an inflammatory periodontal pocket is associated with a reduction in commensal oral bacteria and an expansion of keystone pathobiont bacterial species, such as *Porphyromonas gingivalis*, *Fusobacterium nucleatum*, *Prevotella spp.*, *Campylobacter rectus*, *Parvimonas micra*, *Tanerella forsythensis* and *Tanerella denticola* [11,12]. Globally, PD affects about 20%–50% of the population [13]. Developing nations have higher prevalence of PD among adolescents than developed countries with proportions ranging from 35% to 70% [14,15]. Smoking/tobacco use, obesity, poor oral hygiene and nutrition have all been associated with an increased risk of PD [16,17]. PD prior to and during pregnancy has been associated with a two- to four-fold risk of preterm birth (PTB) [18,19,20], a leading cause of infant mortality and morbidity worldwide [21,22].

Similarly, bacterial vaginosis (BV) is a clinical syndrome characterized by microbial shifts away from the optimal community, which has also been associated with increased risk of PTB [18,19,20]. BV is the most common urogenital disorder of reproductive age women, with prevalence ranging from 25%–50% in different populations [23]. BV is a dysbiosis characterized by a reduction of *Lactobacillus* communities and an expansion of both strictly and facultative anaerobic Gram-negative genera, including *Gardnerella*, *Atopobium*, *Mobiluncus* and *Prevotella* [24,25]. In a study of 296 reproductive-age American women, Ravel et al. (2011) identified five distinct vaginal community state types (CSTs) dominated by *L. crispatus*, *L. gasseri*, *L. iners*, *L. jensenii* and non-lactobacilli bacteria, respectively [26]. African women in particular, including African-American women, appear to be more likely to have a high diversity vaginal microbiota and low relative abundance of *L. crispatus* compared to non-African women [27,28,29,30].

Despite strong epidemiological evidence for a relationship between BV, PD and PTB [18,19,20,31], it is unknown whether microbial dysbiosis in the oral and vaginal cavity are causally linked or whether they are independent risk factors for PTB. Few studies have investigated a possible biological relationship between the oral and vaginal microbiotas [32,33]. Furthermore, the difficulty in diagnosing and treating both oral and vaginal microbial dysbiosis has yielded mixed results of clinical trials using PD and BV treatment to reduce adverse birth outcomes [31]. Here, we aimed to characterize the oral microbiota in a cohort of South African adolescents with a high prevalence of BV and to investigate the potential association between oral bacterial diversity and vaginal microbial dysbiosis.

## 2. Materials and Methods

### 2.1. Study Cohort

Adolescents were recruited through a parent study, UChoose, an open-label, randomized crossover study designed to evaluate the feasibility of different hormonal contraception (HC) options among adolescents (clinicaltrials.gov/NCT02404038) [Gill, K. et al., An Open-Label, Randomized Crossover Study to Evaluate the Acceptability and Preference for Contraceptive Options in Female Adolescents, 15–19 Years of Age, as a proxy for HIV prevention methods (UChoose), *JIAS*, under review]. Approval was obtained from the Human Research Ethics Committee at the University of Cape Town (HREC 801/2014). Participants were screened, those 18 years or older provided informed consent, while informed assent from the participant and informed consent from a parent or legal guardian were obtained for participants younger than 18 years old. Eligibility criteria are described in detail elsewhere [34] [Gill, K. et al., An Open-Label, Randomized Crossover Study to Evaluate the Acceptability and Preference for Contraceptive Options in Female Adolescents, 15–19 Years of Age, as a proxy for HIV prevention methods (UChoose), *JIAS*, under review]. In brief, the eligibility criteria for enrolment for the parent study included either HC naive or willingness to change method, no symptomatic sexually transmitted infections (STIs) within the prior 40 days, no known sensitivity to any of the study products and no intentions of becoming pregnant throughout the study period. For this sub study, samples from baseline were included for participants that met the eligibility criteria, provided both vaginal and oral samples and were diagnosed as BV positive or negative by Nugent scoring.

### 2.2. Sample Collection

At all study visits, a rapid HIV and a pregnancy test were performed and if positive, the participant was counselled and referred for management and no further mucosal samples were collected. A detailed interviewer-assisted questionnaire assessing medical history, sexual behaviour, last menstrual cycle, adherence to study product, intra-vaginal practices, adverse experiences and antibiotic use was completed. The following genital tract samples were collected—two vulvo-vaginal swabs for sexually transmitted infection (STI) testing, Nugent scoring, *Candida* screening and pH measurement and a lateral wall swab for 16S rRNA gene sequencing. Two oral samples were collected in order to sample both the supragingival and salivary bacterial composition. Participants were required to have refrained from eating or drinking anything other than water for at least 30 minutes prior to oral sample collection. Sterile toothpicks were used to sample interdental (incisor-canine) supragingival plaque and then placed into an Eppendorf tube with 500 μL Tris-ethylenediaminetetraacetic acid (EDTA) buffer (10 mM Tris HCl, 1 mM EDTA; pH 8.0). The Eppendorf tube was stored upright at room temperature during transport. Upon arrival in the laboratory, the supragingival samples were vortexed and the toothpicks discarded prior to storage at −80 °C. Saliva samples were collected using the Salivette^®^ (Sarstedt) collection device. Participants were required to chew the swab from the Salivette for 60 s in order to stimulate saliva production. The swab was then spit back into the collection tube and stored upright at 4 °C during transport. Upon arrival at the laboratory the Salivette^®^ tubes were centrifuged at room temperature for 5 min at 4000 rpm. The Salivette swab was discarded and the saliva filtrate was transferred into a 2 mL cryovial. Saliva samples were then centrifuged for 15 min centrifugation at 4000× *g* at 4 °C to pellet cells. Leaving 100 μL, saliva supernatant was removed and stored at −80 °C. The pellet was resuspended in the remaining 100 μL of supernatant and 300 μL Tris-EDTA (pH 8.0) buffer. The resuspended saliva pellets and supragingival samples were then boiled at 95 °C for ten minutes. All samples were stored at −80 °C prior to extraction.

### 2.3. STI and BV Testing

Molecular testing for the following STIs—*Chlamydia trachomatis*, *Neisseria gonorrhoeae*, *Trichomonas vaginalis* and *Mycoplasma genitalium* by multiplex polymerase chain reaction (PCR) was performed as described [35]. If any of these laboratory-based tests were positive, appropriate targeted therapy was prescribed and recorded. Blood was obtained for HIV rapid test and herpes simplex virus 2 (HSV-2) serology. A vulvo-vaginal swab was collected for BV testing (Gram staining and Nugent scoring; BV negative (Nugent 0–3), intermediate (Nugent 4–6) or positive (Nugent 7–10)) and microscopy for *Candida* hyphae and spores. Vaginal pH was measured using colour-fixed indicator strips (Macherey-Nagel, Düren, Germany).

### 2.4. Amplification and Sequencing of the V4 Region of the 16S rRNA Gene

Two oral samples (saliva and supragingival plaque) and a vaginal lateral wall swab were collected for microbiome analysis using 16S rRNA gene sequencing. The oral samples and vaginal swabs were thawed and treated with an enzyme cocktail consisting of mutanolysin (25kU/mL, Sigma Aldrich, St. Louis, MO, USA), lysozyme (450 kU/mL, Sigma Aldrich) and lysostaphin (4 kU, Sigma Aldrich) for 1 h at 37 °C. Microbial DNA was extracted using the *Quick*-DNA^TM^ Fungal/Bacterial Miniprep kit (Zymo Research, Irvine, CA, USA) following the manufacturer’s protocol. Mechanical disruption was performed in a Qiagen TissueLyser LT for 5 min at 50 oz. The V4 hypervariable region of the bacterial 16S rRNA gene was amplified by PCR using modified universal primers [36]—515F (5′- TCG TCG GCA GCG TCA GAT GTG TAT AAG AGA CAG NNN NNG TGC CAG CMG CCG CGG TAA -3′) and 806R (5′- GTC TCG TGG GCT CGG AGA TGT GTA TAA GAG ACA GNN NNN GGA CTA CHV GGG TWT CTA AT -3′). Samples were purified using Agencourt AMPure XP beads (Beckman Coulter, Brea, CA, United States) and quantified using the Qubit dsDNA HS Assay (Life Technologies, Carlsbad, CA, USA). Illumina sequencing adapters and dual-index barcodes were added to the purified amplicon products using limited cycle PCR and the Nextera XT Index Kit (Illumina, San Diego, CA, USA). Amplicons from 96 samples and controls were pooled in equimolar amounts and the resultant libraries purified by gel extraction (Qiagen, Hilden, Germany) and quantified using the Qubit dsDNA HS Assay Kit (Life Technologies). Negative controls included DNA extraction controls using only reagents from the DNA extraction kit and negative water controls for both first and second rounds of PCR. The libraries were sequenced on the Illumina MiSeq platform (300 bp paired-end) with v3 chemistry.

### 2.5. Bioinformatics Analysis of the 16S rRNA Gene Sequencing Data

De-multiplexed, raw reads were pre-processed using usearch7 and modules included in the QIIME package (Quantitative Insights Into Microbial Ecology, http://qiime.org). The quality of raw reads was assessed using FastQC [37]. Using USEARCH, 250 bp paired-end reads were merged and then quality filtered (merged reads were truncated to 250 bp and reads with error scores larger than 0.1 discarded). Next, sequences were de-replicated whilst recording the level of replication for each sequence. De-replicated sequences were sorted by abundance (highest to lowest) and clustered de novo into operational taxonomic units (OTUs) at 97% similarity using usearch7, which implements a greedy algorithm [38]. Chimeric sequences were detected (against the Gold database) using UCHIME [39] and removed. Individual sequences were assigned to specific OTUs using a 97% similarity threshold. Taxonomic assignment was performed using the Ribosomal Database Project (RDP) classifier (against the Greengenes 13.8 database) at the default confidence level of 0.5 [40]. The representative sequence set was then aligned against the Greengenes 13.8 database using PyNAST [41]. For oral OTUs where species level annotation was not achieved using the previously described method, BLASTn searches were performed in the NCBI 16S ribosomal RNA sequence (bacteria and Archaea) database after excluding all uncultured bacteria. If more than one species mapped to an OTU, OTUs were annotated as follows—Genus species A_species B_speciesC for a maximum of three species. If more than three species mapped to an OTU or there was disagreement between the Greengenes and BLASTn annotation, the FASTA sequence was searched using BLASTn in the Human Oral Microbiome Database (eHOMD) [42] and taxa previously identified from the human oral microbiome were selected. Finally, a dendrogram was constructed using FastTree to relate OTUs from the multiple sequence alignment [43]. Based on rarefication analysis for optimal read count depth, samples with > 2000 reads were selected for downstream analyses.

### 2.6. Statistical Analysis

All downstream statistical analysis was performed in R. Differences in study population characteristics were tested using Pearson´s Chi-squared test or Fisher’s exact test (when the expected value was < 5) for count data. Paired or unpaired Student’s t-test was used to test differences in mean (parametric data) and unpaired Mann-Whitney U was applied for differences in medians (non-parametric data). Correlations between continuous data was analysed using Spearman’s correlation. Ecological diversity was calculated using the phyloseq [44] package, cluster [45] was used for community type clustering, vegan [46] for ordinations and redundancy analysis, Non-negative Matrix Factorization (NMF) [47] for annotated heatmaps and DESeq2 [48] for differential abundance testing. Beta diversity non-metric dimensional scaling (NMDS) was performed using Unifrac distances. The Procrustes function from the vegan R package was used to compare the NMDS plots between the oral sites and the vagina with 999 permutations. Species richness boxplots were generated using Chao1 Index and the Kruskal Wallis non-parametric variance test. OTU tables were then standardized (i.e., transformed to relative abundance and multiplied by the median sample read depth) and filtered so that each OTU had at least 10 counts in at least 10% of samples. Vaginal microbial community state types (CSTs) were determined by fuzzy clustering with optimal k (k = 3) using weighted-Unifrac distances as previously described by Lennard et al., 2018 [29]. The overall difference in microbial composition between groups was determined by permutational multivariate analysis of variance (PERMANOVA) using distance matrices (unweighted-UniFrac) with 999 permutations using the adonis2 function; homogeneity of variance between groups was assessed using the betadisper function using vegan [46]. For heatmap and relative abundance plots, OTUs were merged at the lowest available taxonomic level using a custom script developed by Lennard et al., 2018 [29] (https://gist.github.com/kviljoen/97d36c689c5c9b9c39939c7a100720b9). Heatmaps were constructed using weighted-Unifrac as a distance metric and complete unsupervised hierarchical clustering. Differential abundance testing was performed using DESeq2 using a predetermined level of significance (adjusted *p*-value < 0.05).

### 2.7. Data Availability

Raw sequence data for 16S rRNA gene amplicon sequences are available at http://www.ebi.ac.uk/ under project number PRJEB34895. R analysis scripts and additional de-identified metadata available upon reasonable request.

## 3. Results

### 3.1. Cohort Characteristics and Vaginal Microbiota

The cohort has been described in detail previously [Gill, K. et al., An Open-Label, Randomized Crossover Study to Evaluate the Acceptability and Preference for Contraceptive Options in Female Adolescents, 15–19 Years of Age, as a proxy for HIV prevention methods (UChoose), *JIAS*, under review]. Bacterial DNA was extracted from vaginal lateral wall (LW), saliva (SAL) and supragingival plaque (SGP) samples of 94 participants for which all three sample types were available and who had a Nugent score of 7–10 (BV positive) or 0–3 (BV negative). The mean age of these 94 participants was 17 years (standard deviation [sd] = 1.37 (Table 1)). Age, body mass index [BMI], number of previous pregnancies and laboratory diagnosis of at least one bacterial/parasitic sexually transmitted infection (STI) were similar amongst BV positive and BV negative participants (Table 1). *Chlamydia trachomatis* was the most prevalent STI at 35.1%, followed by Herpes Simplex Virus 2 (HSV-2) seropositivity at 33.3%, *Neisseria gonorrhoeae* at 12.8%, *Trichomonas vaginalis* at 6.4% and *Mycoplasma genitalium* at 4.3%. The prevalence of *C. trachomatis*, *T. vaginalis*, *N. gonorrhoeae*, *M. genitalium* and HSV-2 was similar regardless of BV status (Table 1). HC use was common in this cohort but did not differ by BV status.

From these 94 participants, a total of 282 DNA samples were evaluated (including matched LW, SAL and SGP). After QIIME quality filtering, four SAL and two SGP samples were excluded (276/282). Furthermore, all samples with read counts below 2000 were excluded (N total = 18, LW = 1, SAL = 15, SGP = 2). This resulted in a total of 258 samples being included with at least one LW, SAL or SGP sample originating from the 94 participants of which 72 had all three samples available for analysis. In accordance with previously published research [26,30], participants with BV determined by Nugent scoring presented with a diverse vaginal microbiota comprised of high relative abundances of BV-associated bacteria including *Gardnerella*, *Prevotella*, *Lachnovaginosum* (BVAB-1), *Aerococcus*, *Megasphaera*, *Sneathia* and *Atopobium* (Figure S1). In contrast, BV negative participants presented with a vaginal microbiota dominated by *Lactobacillus* spp. (Figure S1). Similar to other African cohorts [29,49], no clusters dominated by *L. gasseri* or *L. jensenii* were identified in this cohort. Instead, three distinct vaginal CSTs—termed CST-I (*L. crispatus* dominated), CST-III (*L. iners* dominated) and CST-IV (diverse, BV-associated bacteria)—were identified [Balle, C. et al., Hormonal contraception alters vaginal microbiota and cytokines in South African adolescents in a randomized trial, *Nat. Commun.*, under revision] (Figure S1). Of the 93 LW samples that were retained after quality assessment, 51.8% (N = 49) belonged to CST-IV, while the remainder of the participants were evenly distributed between CST-I (24.5%, N = 23) and CST-III (23.4%, N = 22) (Figure S1). The majority of the participants (91.8%) with a CST-IV vaginal microbiota had BV at the time of sample collection. In contrast, none of those with CST-I were BV positive by Nugent scoring and only one (4.5%) of those with CST-III were BV positive.

### 3.2. Salivary and Supragingival Oral Microbiota in South African Adolescents

**Figure 1:**
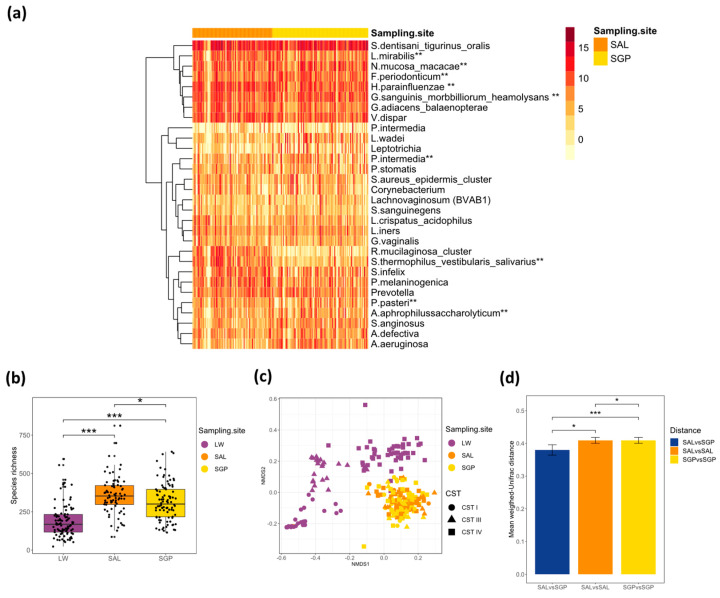
Bacterial composition of the oral microbiota. (**a**) Heatmap showing the relative standardised read counts of the 30 top most abundant bacterial taxa in oral samples (N = 165) clustered by sampling site (SAL: saliva, SGP: supragingival plaque) with log2 colour scale indicating the relative read count of different bacterial taxa in each sample and annotation bar colour key denoting the sample site (orange for saliva (SAL, N = 75) and yellow for supragingival plaque (SGP, N = 90). (**b**) Boxplot depicting the species richness of oral and vaginal samples by Chao1 and (**c**) Non-metric Multi-dimensional Scaling (NMDS) plots depicting the beta diversity of the oral and vaginal microbiota using weighted-Unifrac as a distance metric. Shapes depict vaginal community state types (CSTs). (**d**) Bar plots depicting the mean weighted-Unifrac distance between the salivary (SAL) and supragingival (SGP) microbiota within participants (SALvsSGP) and between participants according to either SAL (SALvsSAL) or SGP microbiota (SGPvsSGP). ** Species annotation with lower than 97% identity using BLASTn to search the expanded Human Oral Microbiome Database (eHOMD) database.

A total of 775 operational taxonomic units (OTUs) were present in at least 10% of all oral samples at a relative abundance higher than 0.01%. Of these, 560 were ubiquitous across both oral sampling sites, 101 were present in only SGP (N = 90) and 114 in only SAL (N = 75) samples. For the description of the core oral microbiota, OTUs present in at least 50% of samples in both SAL and SGP across all participants with a relative abundance greater than 0.01% were included. The core oral microbiota was dominated by Firmicutes (41%), followed by Proteobacteria (26%), Bacteroides (18%), Fusobacteria (9%) and Actinobacteria (6%) (Table 2). On a genus level, the core oral microbiota was comprised of *Streptococcus* [*S. dentisani_tigurinus_oralis* (29%), *S. anginosus* (1%)], *Haemophilus* [*H. parainfluenzae* (8%), *H. influenzae* (1%)], *Prevotella* [*P. melaninogenica* (3%), *P. intermedia* (2%), *P. oris* (1%)], *Neisseria* [*N. mucosa_macacae* (5%)] and *Veillonella* [*V. dispar* (5%)]. All other genera were only present at a relative abundance < 5% (Table 2, Figure 1a).

For 17 participants, after quality filtering, the only oral samples available for analysis of the oral microbiome was the SGP sample. Two participants had only the SAL oral sample available. This resulted in 73 participants having both SAL and SGP samples and data available for analysis. There was a significant difference in the species richness (measured using Chao1) between paired SAL and SGP samples (N = 146), with the salivary microbiota being more diverse (median Chao1 (IQR)—354 (295–420) vs. 317 (260–404)), *p* = 0.030; Figure 1b). However, the species richness of the SAL and SGP within participants were weakly correlated (R = 0.22, *p* = 0.068; data not shown). Permutational multivariate analysis of variance (PERMANOVA) of the weighted-Unifrac distances between matched oral samples revealed significant separation of the salivary and supragingival microbiota (*p* = 0.001, R2 = 0.038, Figure 1c). Furthermore, the within-participant variability (between oral sites) in beta diversity was significantly less than the variability of samples from same site between different participants based on mean weighted-Unifrac distances (73 participants, SAL: *p* = 0.015, SGP: *p* < 0.001; Figure 1d). Beta diversity variability was also significantly higher in the SGP than SAL samples (*p* = 0.024; Figure 1d). Using DESeq2, we found several significantly differentially abundant taxa between SAL and SGP samples, including Firmicutes such as *Oribacterium* spp. (O. *asaccharolyticum* and *O. sinus*), *Streptococcus thermophilus_vestibularis_salivarius* and *Selemonas infelix*, Bacteroidetes such as *Porohyrpmonas* spp. (*P. endotalis* and *P. pasteri*) and *Prevotella* spp. (*P*. *nanciensis*, *P*. *aurantiaca* and *P*. *oris*), which were more abundant in SAL samples (Table S1). Fusobacteria belonging to the *Leptotrichia* genus (*L. buccalis_trevisanii*, *L. wadei* and *L. hofstadii*) and Actinobacteria belonging to the *Actinomyces* genus (*A. aeruginosa*, *A. israelii* and *A. dentalis*) were more abundant in the SGP samples (Table S1), as were the PD-associated species *F. nucleatum*, *P. intermedia* and *Tanerella forsythensis*. As diet may influence the oral microbiota, we tested if there was any association with body mass index (BMI). There were no significant correlations between the species richness of the SAL or SGP microbiota and the BMI of the participants (SAL: R = 0.089, *p* = 0.450; SGP: R = 0.100, *p* = 0.360).

### 3.3. Relationship between the Oral and Vaginal Microbiota

**Figure 2:**
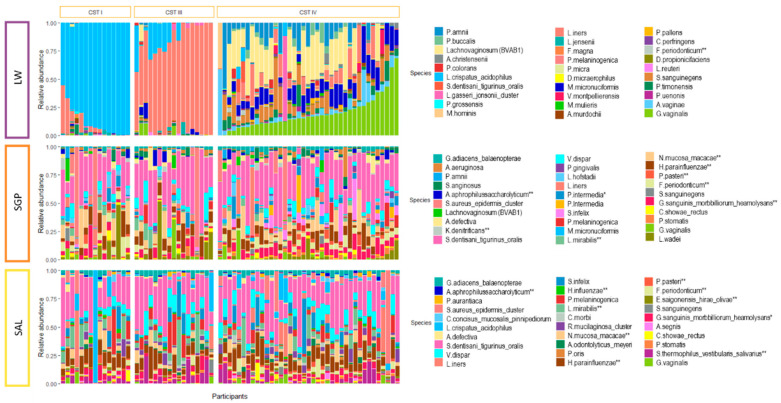
Microbial communities in the vagina, saliva and supragingival space. Bar plot depicting the relative abundance of the 30 most abundant bacterial species for each site in matched vaginal (LW, top), supragingival (SGP, middle) and salivary (SAL, bottom) samples identified by 16S rRNA microbiome profiling. The samples are aligned by PID (N = 72) and grouped by vaginal community state type (CST) (CST-I, CST-III, CST-IV) established using soft k-means clustering with weighted-UniFrac distances and ordered based on the abundance of the most dominant species in each CST (CST-I: *L. crispatus*, CST-III: *L. iners* and CST-IV: *G. vaginalis*).

We next evaluated the relationship between oral and vaginal microbiota (Figure 2). The oral and vaginal bacterial communities of South African adolescent females were distinct in terms of bacterial composition, species richness and beta diversity (Figure 1b,c, Figure 2). The median alpha diversity of both the SAL and SGP samples was significantly higher than that of vaginal samples (LW: median Chao1 (IQR) 168 (117-428)) (*p* < 0.001, Figure 1b). 

**Figure 3:**
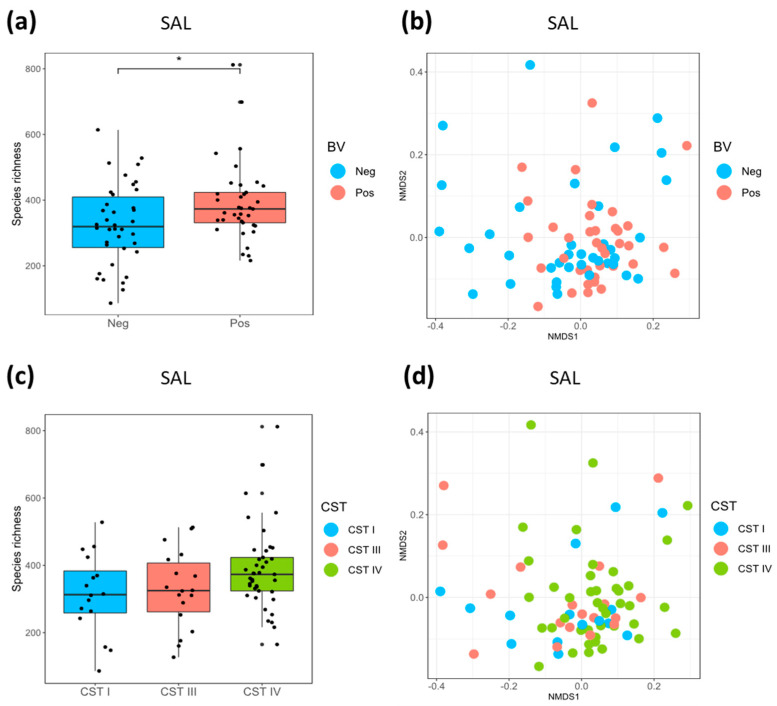
Species richness and beta diversity of saliva samples by BV status and CST. Boxplots showing species richness measured using Chao1 of saliva (SAL) samples according to (**a**) bacterial vaginosis (BV) status (Neg = deepskyblue, Pos = salmon) and (**c**) vaginal community state type (CST) (CST-I = blue, CST-III = salmon, CST-IV = green). Non-metric Multi-dimensional Scaling (NMDS) plots showing beta diversity of saliva (SAL) (N = 75) samples calculated using unweighted-Unifrac distances according to (**b**) BV status and (**d**) vaginal CSTs.

The species richness of the SAL microbiota was higher in BV positive compared to BV negative women (median SAL Chao1 (IQR) 373 (331–585) versus 319 (256–515), *p* = 0.030; Figure 3a). This was also true for women assigned to CST-IV (median SAL Chao1 (IQR) 373 (324–614)) compared with CST-I (median SAL Chao1 (IQR) 313 (258–474), *p* = 0.076) and CST-III (median SAL Chao1 (IQR) 325 (262–510), *p* = 0.107; Figure 3c) although not significantly so. In contrast, there were no differences in species richness of SGP samples according to vaginal CSTs or BV status (Figure S3). There was a significant difference in beta diversity measured using unweighted-Unifrac distances of SAL samples based on BV status (*p* = 0.033, R2 = 0.024) (Figure 3b) and a trend towards a significant difference between CST-IV and CST-I (*p* = 0.062, R2 = 0.028). Beta diversity differences of the SAL microbiota were not evident between CST-IV and CST-III (*p* = 0.116, R2 = 0.023) nor CST-I and CST-III (*p* = 0.992, R2 = 0.017; Figure 3d).

No differences in beta diversity were found for the SGP microbiota between BV positive or negative participants (*p* = 0.416, R^2^ = 0.013) or according to CST (*p* = 0.187, R^2^ = 0.027) (Figure S3). For participants with paired oral and vaginal data available, we furthermore tested the fit of the multidimensional shapes (NMDS ordinations) between the different sites using Procrustes analysis (Figure S2). There was a significant correlation between the two oral site ordinations (SGP versus SAL, *p* = 0.006, m^2^ = 0.8892) and between the vaginal and the SAL ordinations (*p* = 0.024, m^2^ = 0.9391). However, the measure of fit between the ordinations (m^2^) were high, suggesting that the correlation may be weak despite their statistical significance. There was no correlation between the vaginal and SGP ordinations (*p* = 0.733, m^2^ = 0.9902).

*Prevotella intermedia*, a taxon associated with both PTB and PD [50], was significantly more abundant in SGP of participants with the diverse vaginal CST-IV versus both lactobacilli dominated vaginal CSTs using DESeq2 (Table 3 (A,B) (adjusted *p* < 0.05). Another PD-associated bacteria, *Porphyromonas endodontalis*, was also more abundant in the SGP microbiota of participants with CST-IV versus CST-I (Table 3 (B); adjusted *p* = 0.041). When comparing the composition of the salivary microbiota of this cohort with respect to CST (Table 3 (A,B)), *Prevotella intermedia* and *Pseudomonas aerugonosa* were both less abundant in participants with *L. crispatus* dominated CST-I versus CST-IV but only *Pseudomonas aeruginosa* remained significant after adjusting for multiple comparisons (Table 3 (A)). There were no differentially abundant taxa between SAL of participants with a CST-I and CST-III vaginal community (Table 3 (D)). Although there were some significantly differentially abundant, non-PD-associated bacteria in all comparisons, we did not identify any differences in the relative proportion of other PD-associated bacteria (*Porphyromonas gingivalis*, *Fusobacterium nucleatum*, *Prevotella nigrescens*, *Campylobacter rectus*, *Parvimonas micra*, *Tanerella forsythensis* and *Tanerella denticola*) in oral microbiota of participants with and without BV or different CSTs that were significant (Table 3 (A–D)).

## 4. Discussion

While a relationship between BV, PD and PTB has been suggested by strong epidemiological evidence [18,19,20,31], very few studies have investigated a possible biological relationship between oral and vaginal microbiotas [32,33] and none have investigated the oral microbiota in the context of active BV cases or non-optimal vaginal CSTs. In the present study, we found that the salivary microbiota of participants with BV was significantly more diverse than in participants with lactobacillus dominated communities. Participants with diverse vaginal CST (CST-IV) also had a more diverse salivary community albeit this was not statistically significant potentially due to sample size. Furthermore, BV explained a significant portion of the between community distance of the SAL microbiota. Although the species richness of the SGP microbiota was not strongly associated with the vaginal microbiota, several PD-associated bacteria including *Prevotella intermedia* and *Porphyromonas endodontalis* were found to be more abundant in the SGP microbiota of participants with a dysbiotic vaginal community as determined by molecular methods (CSTs). For the salivary microbiota, *Prevotella intermedia* and *Pseudomonas aeruginosa* were also found to be enriched in participants with vaginal dysbiosis. These results corroborates data from a two US studies looking at the association of BV with PD [32] and gingivitis [33], a condition that is often a precursor to PD, which found that women with a BV (Nugent score ≥ 7) were more likely to have gingivitis and PD [32,33]. Furthermore, independently of BV, women with gingivitis had higher vaginal bacterial counts of the *Prevotella* species *P. bivia* and *P. disiens* (*p* < 0.001) [33]. Together, these data support a relationship between the oral and vaginal microbiota.

Herein, we also give a description of and compare the salivary and supragingival microbiotas of South African adolescent females. In line with what has been previously published in other populations [1,2,4], we found the core oral microbiota to be dominated by Firmicutes and Proteobacteria. As expected, *Streptococcus* was the most prevalent bacterial genus, followed by *Haemophilus*, *Neisseria* and *Veillonella*. When comparing the salivary and supragingival microbiota to each other, we found that the majority of oral OTUs were present in both communities and that there was a weak but positive correlation of the species richness between the two sites. Also, the beta diversity between sites within participants was less variable than for samples from each site between different participants, suggesting that the bacterial composition of the two sites are more similar within than between individuals. Yet, the species richness of the salivary microbiota was significantly more diverse than the SGP microbiota and these two sites communities clustered distinctly. These microbial differences observed between the two oral sites could partly explain why we observed different degrees of relationship between each oral site and vaginal dysbiosis with the salivary microbiota being more strongly related. The salivary microbiota may be more responsive to factors which also affect bacteria in the vagina compared to the supragingival plaque, which may be more resistant to change. The bacteria in the saliva may also be more likely found in the gastrointestinal tract and thus more closely associated with potential bacterial translocation to the genital tract and anal-vaginal transfer of bacteria.

The extent to which host genetics and environmental factors determine the composition of the oral microbiota is not well understood. While one study found that the salivary microbiota did not vary across individuals from twelve locations worldwide [51], another study comparing saliva from Alaskan, German and North African individuals found significantly higher bacterial diversity in the oral microbiotas of Africans compared to those from Northern countries [52]. Similarly, in the vaginal compartment, high diversity vaginal bacterial communities not dominated by *Lactobacillus* species have been more commonly found in African women compared to Caucasians women [26,53,54,55]. Together, these data could suggest that factors that affect the oral and the vaginal microbiota could be linked.

During pregnancy, microbial dysbiosis in the oral and vaginal tract are thought to initiate a host and/or foetal immune response [31]. Bacteria are hypothesized to access the uterine cavity by ascending through the genital tract or via systemic entry points, such as the oral cavity, triggering a clinical or subclinical inflammatory cascade that results in the release of proinflammatory cytokines and prostaglandins, inducing myometrial contractions and spontaneous PTB [31]. It is currently unknown to what extent this is a localized inflammatory response as the result of colonization of bacteria from distal sites to the uterine tissue or a systemic inflammatory response triggered by bacterial dysbiosis at distal sites. A better understanding of how differences in host genetics could potentially determine the composition of both the vaginal and oral microbiota and how this in turn impacts the inflammatory response on a local or systemic level, is required to disentangle the relationship between BV, PD and PTB.

One limitation to this study was that we were unable to establish oral health (defined by PD diagnosis) or oral health practices in the cohort, at the time of sample collection. In addition, we did not have information on behavioural factors, including diet and tobacco use, which are known to increase risk of PD [16,17]. Yet, BMI was not associated with the diversity of the oral microbiota or BV status. Other factors that may impact the oral microbiota and BV status include use of antibiotics and hormonal contraception. However, there was minimal use of antibiotics in this cohort prior to sampling and was therefore not assessed further. Conversely, there was a high level of hormonal contraceptive use in this cohort, albeit evenly distributed between BV negative and positive participants (83% and 71%, respectively). Use of hormonal contraception has been shown to alter vaginal microbiota composition [Balle, C. et al., Hormonal contraception alters vaginal microbiota and cytokines in South African adolescents in a randomized trial, *Nat. Commun.*, under revision] [56,57,58]. Hormonal contraceptive use, including the injectable depot-medroxyprogesterone acetate (DMPA) and combined oral contraceptives, have similarly been implicated in changes in periodontal conditions and increased gingival inflammation in observational cohorts [59,60,61,62,63,64]. Furthermore, a higher prevalence of PD-associated species has been found in women using combined oral contraceptives compared to non-contraceptive users [59,60,64]. A deeper evaluation of the potential impact of hormonal contraceptive use on the oral microbiota is thus warranted.

Our inability to distinguish between certain bacterial species, including key species such as those of the genera *Streptococcus*, which comprise the majority of the oral microbiota, represent a limitation of this study and impacted our ability to determine the functional and clinical relevance of the distinct OTUs we observed. Targeted qPCR or a larger 16S gene fragment would assist in resolving the species level classification and should be a consideration for future work.

This study provides evidence to support a relationship between oral and vaginal dysbiosis, although the causal relationship could not be determined. Future studies that assess the relationship between PD and BV could lead to potential screening and intervention programs to effectively identify and treat risk factors during pregnancy and reduce the amount of preventable maternal and infant deaths in Sub-Saharan Africa.

## Figures and Tables

**Table 1:** Cohort characteristics by bacterial vaginosis (BV) status.

	BV Status		
	Negative (N = 48) (Nugent 0–3)	Positive (N = 46) (Nugent 7–10)	*P* Value
Mean Age (Std. Deviation)	16.9 (1.38)	17.1 (1.36)	0.368
Mean BMI (Std. Deviation)	25.9 (4.67)	26.3 (5.55)	0.710
Previous Pregnancy ^†^	10.6% (N = 4)	13.0% (N = 6)	0.970
Any bacterial STI	43.8% (N = 21)	47.8% (N = 22)	0.850
*Chlamydia trachomatis*	33.3% (N = 16)	37.0% (N = 17)	0.879
*Neisseria gonorrhoeae*	10.4% (N = 5)	15.2% (N = 7)	0.698
*Trichomonas vaginalis*	6.3% (N = 3)	6.5% (N = 3)	1.000
*Mycoplasma genitalium*	4.2% (N = 2)	4.3% (N = 2)	1.000
Herpes Simplex Virus 2	31.9% (N = 15)	34.8% (N = 16)	0.942
Any medication past month	20.8% (N = 10)	10.6% (N = 5)	0.300
Any antibiotic use past month	4.2% (N = 2)	8.9% (N = 4)	0.634
Current hormonal contraception ^‡^	83.0% (N = 39)	71.1% (N = 32)	0.268
Intra-vaginal practices ^§^ Wash vagina with water Douching	4.4% (N = 2) 0.0% (N = 0)	17.1% (N = 7) 0.4% (N = 1)	0.080 0.477
Community state type (CST) CST-I CST-III CST-IV	47.9% (N = 23) 43.8% (N = 21) 8.3% (N = 4)	0.0% (N = 0) 2.2% (N = 1) 97.8 (N = 45)	**<0.001**
Median supragingival plaque (SGP) richness ^¶^ (IQR)	277 (215-525)	324 (240-522)	0.294
Median salivary (SAL) richness ^¶^ (IQR)	319 (256-515)	373 (331-585)	**0.030**
Vaginal samples included	47	46	
Saliva (SAL) samples included	38	37	
Supragingival plaque (SGP) samples included	46	44	

Chi-squared test (Fisher’s exact test when expected values < 5) for the assessment of association of frequency among groups, unpaired Mann–Whitney U test for comparison of medians and unpaired Student’s t test for comparison of means. BMI, body mass index; STI; sexually transmitted infection. ^†^ Missing data from 1 participant (BV negative = 1). ^‡^ Missing data from 2 participants (BV negative = 1, BV positive = 1). ^§^ Missing data from 8 participants (BV negative = 3, BV positive = 5). ^¶^ Chao1.

**Table 2:** Mean relative abundance of core oral taxa merged at lowest annotation level possible.

Phylum	Genus	Species	Mean Relative Abundance
Actinobacteria	*Actinomyces*	*oris_naeslundii _viscosus*	2%
	*Corynebacterium*		2%
	*Gardnerella*		1%
	*Rothia*	*mucilaginosa_cluster*	1%
Bacteroidetes	*Porphyromonas*	*pasteri*	1%
	*Prevotella*	*intermedia*	2%
	*Prevotella*	*melaninogenica*	3%
	*Prevotella*	*oris*	1%
Firmicutes	*Abiotrophia*	*defective*	1%
	*Catonella*	*morbi*	0%
	*Lactobacillus*	*iners*	3%
	*Lactobacillus*	*crispatus*	3%
	*Granulicatella*	*adiacens_balaenopterae*	2%
	*Peptostreptococcus*	*stomatis*	1%
	*Selenomonas*	*infelix*	4%
	*Lachnovaginosum*	*genomospecies (BVAB1)*	1%
	*Staphylococcus*		4%
	*Streptococcus*	*anginosus*	1%
	*Streptococcus*	*dentisani_tigurinus_oralis*	29%
	*Veillonella*	*Dispar*	5%
	*Gemella*	*sanguinis_morbbilliorum_heamolysans*	4%
Fusobacteria	*Fusobacterium*	*periodonticum*	3%
	*Leptotrichia*	*wadei*	3%
Proteobacteria	*Aggregatibacter*	*aphrophilussaccharolyticum*	1%
	*Campylobacter*	*showae_rectus*	2%
	*Kingella*	*dentrificans*	1%
	*Haemophilus*	*parainfluenzae*	8%
	*Haemophilus*	*influenzae*	1%
	*Lautropia*	*mirabilis*	4%
	*Neisseria*	*mucosa_macacae*	5%

**Table 3:** Results of a DESeq2 to assess differences in the relative abundance of supragingival (SGP) and salivary (SAL) bacterial taxa between participants with differing vaginal community state types ((A) CST-IV versus CST-I, (B) CST-IV versus CST-III and (C) CST-I versus CST-III) and (D) participants with and without BV (BV negative as reference).

**A.**				**Vaginal Community State Type (CST)**	
	**Phylum**	**Genus**	**Species**	**CST IV vs. CST-I** **Log2 Fold Change**	***P* Value** **(Adjusted *p*-Value)**
**SGP (N = 69)**	Proteobacteria	*Moraxella*	*NA*	−25.73	2.98 × 10^−28^ (8.31 × 10^−26^)
	Proteobacteria	*Methylobacterium*	*NA*	−6.051	1.15 × 10^−6^ (0.0002)
	Proteobacteria	*Rhizobium*	*leguminosarum*	−5.063	0.0002 (0.0142)
	Bacteroidetes	*Prevotella*	*intermedia*	−3.209	0.0006 (0.0306)
	Proteobacteria	*Delftia*	*acidovorans*	−2.710	0.0005 (0.0306)
	Bacteroidetes	*Porphyromonas*	*endodontalis*	−2.201	0.0009 (0.0414)
	Firmicutes	*Lactobacillus*	*iners*	1.671	0.0017 (0.0606)
	Tenericutes	*Ureaplasma*	*NA*	4.145	0.0017 (0.0606)
	Proteobacteria	*NA*	*NA*	−3.056	0.0034 (0.0868)
	Firmicutes	*Oribacterium*	*NA*	2.301	0.0030 (0.0868)
	Firmicutes	*Blautia*	*NA*	−4.027	0.0032 (0.0868)
**SAL (N = 57)**	Proteobacteria	*Pseudomonas*	*aeruginosa*	−3.716	0.0002 (0.0444)
	Bacteroidetes	*Odoribacter*	*NA*	−3.338	0.0008 (0.0817)
	Firmicutes	*Megasphaera*	*NA*	−3.259	0.0011 (0.0817)
	Proteobacteria	*Moraxella*	*NA*	−3.142	0.0017 (0.0920)
	Bacteroidetes	*Prevotella*	*intermedia*	−3.003	0.0027 (0.0976)
	Actinobacteria	*Mobiluncus*	*mulieris*	−3.015	0.0026 (0.0976)
**B.**				**Vaginal Community State Type (CST)**	
	**Phylum**	**Genus**	**Species**	**CST IV vs. CST III** **Log2 Fold Change**	***P* Value** **(Adjusted *p*-Value)**
**SGP (N = 68)**	Bacteroidetes	*Prevotella*	*intermedia*	−3.471	0.0005 (0.0089)
	Fusobacteria	*Leptotrichia*	*wadei*	3.522	0.0004 (0.0287)
	Proteobacteria	*Delftia*	*acidovorans*	−3.700	0.0002 (0.0287)
	Bacteroidetes	*Prevotella*	*NA*	−3.464	0.0005 (0.0287)
	Firmicutes	*Blautia*	*NA*	−3.189	0.0014 (0.0287)
	Firmicutes	*Clostridium*	*saccharogumia*	−2.918	0.0035 (0.0641)
	Proteobacteria	*Brachymonas*	*denitrificans*	2.903	0.0037 (0.0997)
	Firmicutes	*Oribacterium*	*NA*	2.963	0.0030 (0.0997)
	Proteobacteria	*Morganella*	*NA*	−3.000	0.0027 (0.0997)
**SAL (N = 59)**	Firmicutes	*Staphylococcus*	*aureus_epidermis_cluster*	−3.811	0.0001 (0.0259)
	Bacteroidetes	*Prevotella*	*amnii*	−3.556	0.0004 (0.0273)
	Fusobacteria	*Sneathia*	*sanguinegens*	−3.483	0.0005 (0.0273)
	Firmicutes	*Anaerovorax*	*NA*	−3.439	0.0006 (0.0273)
	Firmicutes	*Megasphaera*	*micronuciformis*	−3.195	0.0014 (0.0522)
	Proteobacteria	*Pseudomonas*	*aeruginosa*	−2.944	0.0032 (0.0758)
	Firmicutes	*Lactobacillus*	*iners*	−3.031	0.0024 (0.0758)
	Firmicutes	*Dialister*	*micraerophilus*	−2.944	0.0032 (0.0758)
	Bacteroidetes	*Prevotella*	*tannerae*	−2.822	0.0048 (0.0944)
	Bacteroidetes	*Bacteroides*	*heparinolyticus*	−2.791	0.0052 (0.0944)
	Firmicutes	*Facklamia*	*NA*	−2.773	0.0056 (0.0944)
	Bacteroidetes	*Prevotella*	*melaninogenica*	2.698	0.0070 (0.0989)
	Firmicutes	*Lactobacillus*	*gasseri_jonsonii_cluster*	−2.678	0.0074 (0.0989)
	Actinobacteria	*Gardnerella*	*vaginalis*	−2.716	0.0066 (0.0989)
**C.**				**Vaginal Community State Type (CST)**	
	**Phylum**	**Genus**	**Species**	**CST I vs. CST III** **Log2 Fold Change**	***P* Value** **(Adjusted *p*-Value)**
**SGP (N = 43)**	Firmicutes	*Staphylococcus*	*aureus_epidermis_cluster*	−2.845	0.0005 (0.0886)
	Bacteroidetes	*Porphyromonas*	*bennonis*	−3.2701	0.0060 (0.0991)
	Firmicutes	*Shuttleoworthia*	*BVAB-1*	−2.201	0.0042 (0.0991)
	Firmicutes	*Clostridium*	*butyricum*	−4.755	0.0014 (0.0991)
	Firmicutes	*Moryella*	*indoligenes*	−4.095	0.0054 (0.0991)
	Proteobacteria	*Kingella*	*denitrificans*	−2.201	0.0038 (0.0991)
	Firmicutes	*Streptococcus*	*thermophilus_vestibularis_salivarius*	1.684	0.0066 (0.0991)
	Bacteroidetes	*Bacteroides*	*fragilis*	−4.210	0.0024 (0.0991)
	Firmicutes	*Dialister*	*propionicifaciens*	−2.856	0.0062 (0.0991)
	Firmicutes	*Megasphaera*	*micronuciformis*	−2.160	0.0034 (0.0991)
	Proteobacteria	*Morganella*	*NA*	−3.839	0.0031 (0.0991)
	Proteobacteria	*Herbaspirillum*	*NA*	4.047	0.0046 (0.0991)
**SAL (N = 34)**	-	-	-	NA	NA
**D.**				**BV Status**	
	**Phylum**	**Genus**	**Species**	**Neg vs. Pos** **Log2 Fold change**	***P* Value** **(Adjusted *p*-Value)**
**SGP (N = 90)**	Proteobacteria	*Delftia*	*acidovorans*	2.437	4.65 × 10^−5^ (0.0066)
	Proteobacteria	*Methylobacterium*	*NA*	3.893	5.39 × 10^−5^ (0.0066)
	Bacteroidetes	*[Prevotella]*	*NA*	1.756	0.0007 (0.0556)
	Bacteroidetes	*Prevotella*	*intermedia*	1.758	0.0023 (0.0618)
	Firmicutes	*Staphylococcus*	*aureus_epidermis_cluster*	−1.734	0.0012 (0.0618)
	Fusobacteria	*Leptotrichia*	*wadei*	−2.043	0.0019 (0.0618)
	Firmicutes	*Lactobacillus*	*crispatus_acidophilus*	−1.487	0.0015 (0.0618)
	Firmicutes	*Oribacterium*	*NA*	−2.208	0.0015 (0.0618)
	Bacteroidetes	*Chryseobacterium*	*NA*	−2.648	0.0022 (0.0618)
	Tenericutes	*Ureaplasma*	*NA*	−3.654	0.0025 (0.0618)
	Proteobacteria	*NA*	*NA*	2.494	0.0036 (0.0762)
	Firmicutes	*Blautia*	*NA*	3.409	0.0037 (0.0762)
	Firmicutes	*Ruminococcus*	*bromii*	3.036	0.0041 (0.0762)
	Proteobacteria	*Neisseria*	*oralis*	−2.362	0.0052 (0.0888)
	Proteobacteria	*Rhizobium*	*leguminosarum*	3.086	0.0054 (0.0888)
	Actinobacteria	*Corynebacterium*	*durum*	−1.530	0.0064 (0.0989)
**SAL (N = 75)**	Fusobacterium	*Sneathia*	*sanguinegens*	1.757	0.00015 (0.0417)

BV, bacterial vaginosis; CST, community state type; SAL, saliva; SGP, supragingival. Results with an adjusted *p*-value of 0.1 included.

## Supplementary Materials

The following are available online at https://www.mdpi.com/2076-2607/8/7/1004/s1, Figure S1: Composition of vaginal bacterial community state types, Figure S2. Comparison of vaginal and oral bacterial communities suing Procrustes analysis of non-metric multidimensional scaling (NMDS) ordination plots, Figure S3. Species richness and beta diversity of supragingival samples by bacterial vaginosis status and community state types, Table S1: Results of DESeq2 analysis for differentially abundant bacterial taxa between supragingival and salivary ecological niches (with supragingival samples as a reference).
