# Supplementary material for: Relationship between the Oral and Vaginal Microbiota of South African Adolescents with High Prevalence of Bacterial Vaginosis
Source: Microorganisms. 2020 Jul 4;8(7):1004. doi: 10.3390/microorganisms8071004 (PMC7409319; doi:10.3390/microorganisms8071004)
Supplement: Supplementary file 1 [file microorganisms-08-01004-s001.zip › microorganisms-827284 suppl for XML conversion/Figure S1.docx]

**
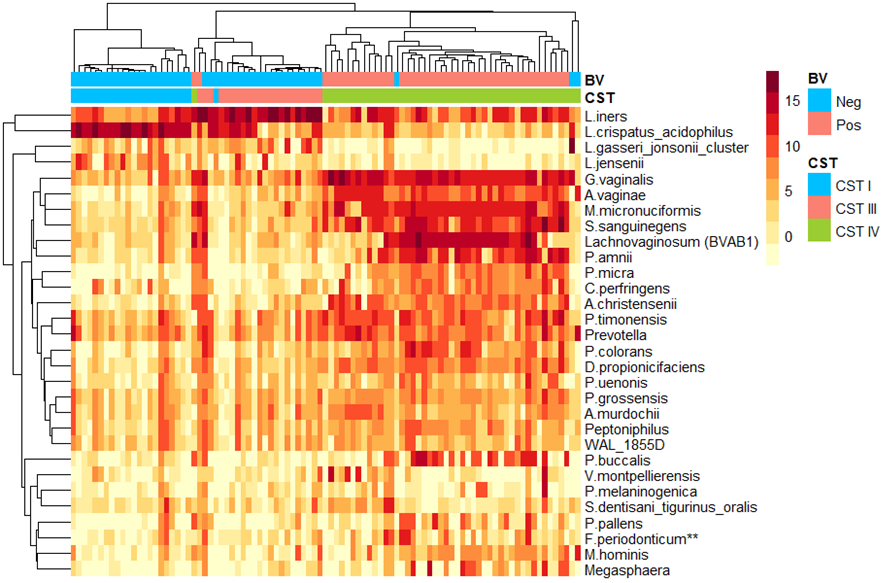
**

**Figure S1.** Composition of vaginal bacterial community state types. Relative standardised read counts of the 30 top most abundant bacterial taxa in vaginal samples (N = 93) clustered using weighted-Unifrac distances and complete hierarchical clustering with log2 colour scale indicating the relative read count of different bacterial taxa in each sample and the annotation bar colour key denoting vaginal community state type (CST) and bacterial vaginosis (BV) status.
