# Supplementary material for: Relationship between the Oral and Vaginal Microbiota of South African Adolescents with High Prevalence of Bacterial Vaginosis
Source: Microorganisms. 2020 Jul 4;8(7):1004. doi: 10.3390/microorganisms8071004 (PMC7409319; doi:10.3390/microorganisms8071004)
Supplement: Supplementary file 1 [file microorganisms-08-01004-s001.zip › microorganisms-827284 suppl for XML conversion/Figure S2.docx]

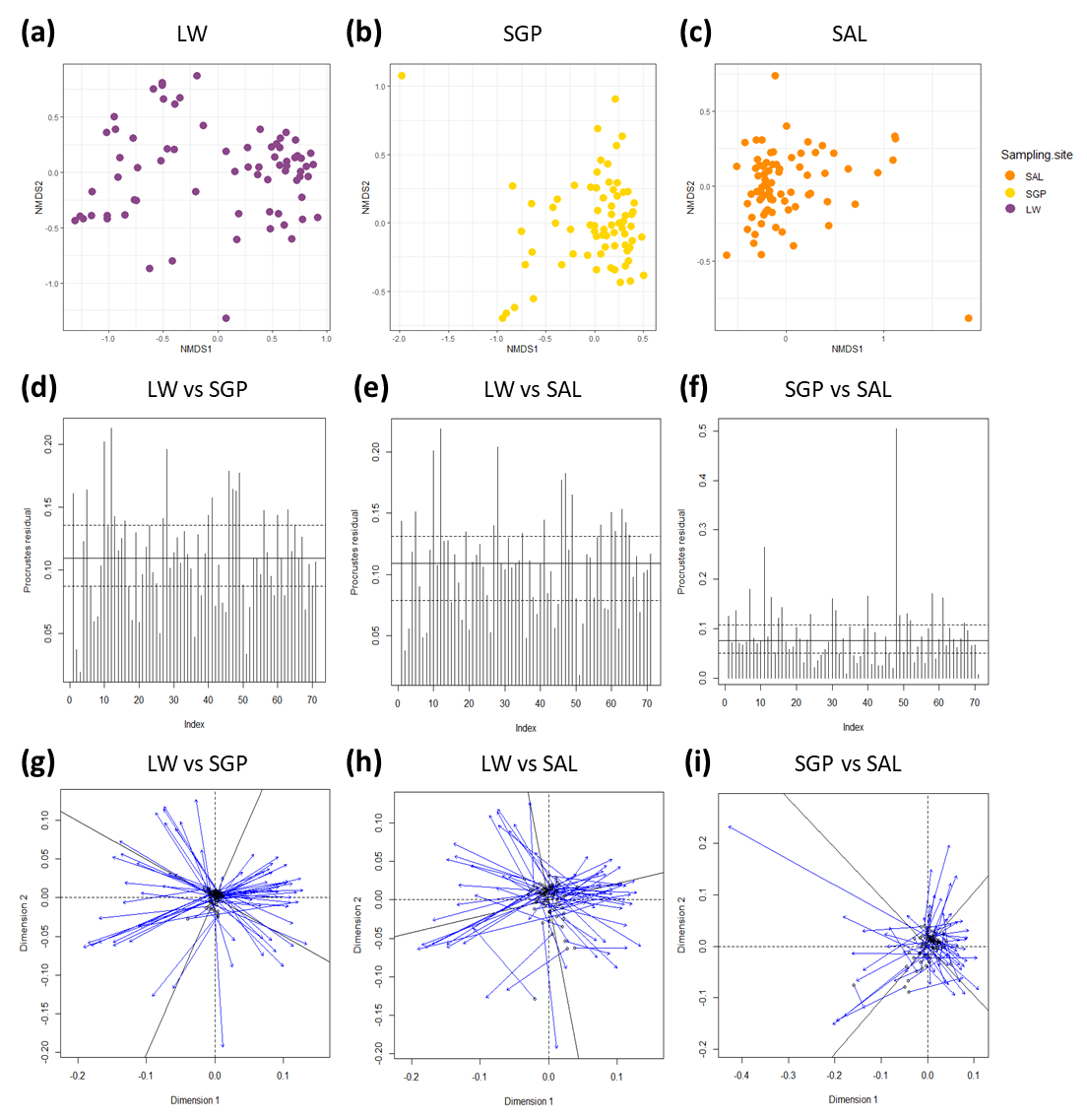


**Figure S2.** Comparison of vaginal and oral bacterial communities suing Procrustes analysis of non-metric multidimensional scaling (NMDS) ordination plots. NMDS plots of the beta diversity of paired (N = 72**)** (**a**) lateral wall vaginal (LW), (**b**) supragingival (SGP) and (**c**) salivary (SAL) microbiota. Procrustes residual plots for comparisons between (**d**) LW and SGP ordinations, (**e**) LW and SAL ordinations and (**f**) SGP and SAL ordinations. The horizontal lines, from bottom to top, are the 25% (dashed), 50% (solid), and 75% (dashed) quantiles of the residuals. Ordination plots indication of the degree of match between (**g**) LW and SGP ordinations, (**h**) LW and SAL ordinations and (**i**) SGP and SAL ordinations. The black points show the position of the samples in the first ordination, and arrows point to their positions in the target ordination. The plots also show the rotation between the two ordinations necessary to make them match as closely as possible.
