# Supplementary material for: Relationship between the Oral and Vaginal Microbiota of South African Adolescents with High Prevalence of Bacterial Vaginosis
Source: Microorganisms. 2020 Jul 4;8(7):1004. doi: 10.3390/microorganisms8071004 (PMC7409319; doi:10.3390/microorganisms8071004)
Supplement: Supplementary file 1 [file microorganisms-08-01004-s001.zip › microorganisms-827284 suppl for XML conversion/Figure S3.docx]

**
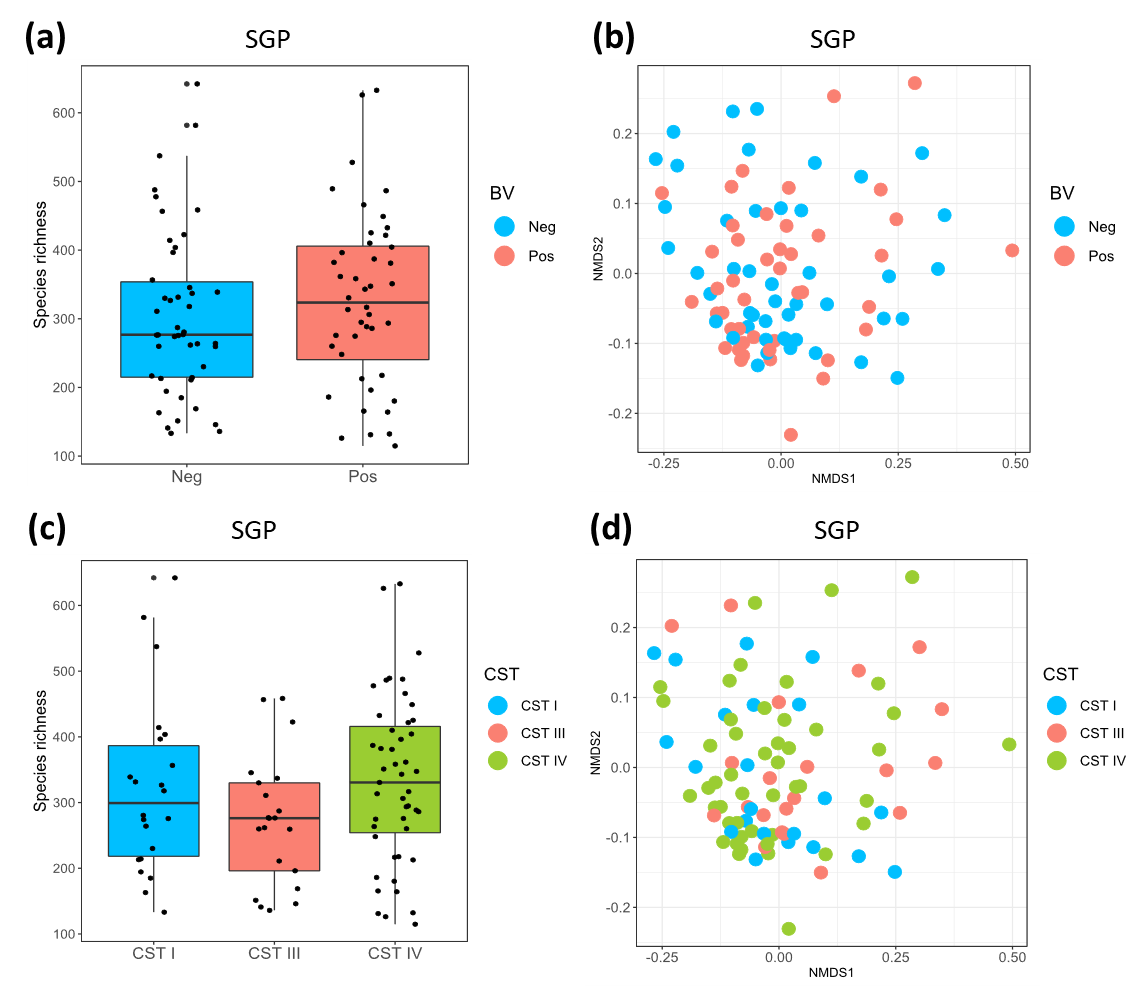
**

**Figure S3.** Species richness and beta diversity of supragingival samples by BV status and CST. Boxplots showing species richness measured using Chao1 of supragingival (SGP) and saliva (SAL) samples according to (**a**) bacterial vaginosis (BV) status (Neg=blue, Pos=salmon) and (**b**) vaginal community state type (CST) (CST-I=blue, CST-III=salmon, CST-IV=green). Non-metric multi-dimensional scaling (NMDS) plots showing beta diversity of supragingival (SGP) (N = 90) samples calculated using unweighted-Unifrac distances according to (**c**) BV status and (**d**) vaginal CSTs.
