# Supplementary material for: Relationship between the Oral and Vaginal Microbiota of South African Adolescents with High Prevalence of Bacterial Vaginosis
Source: Microorganisms. 2020 Jul 4;8(7):1004. doi: 10.3390/microorganisms8071004 (PMC7409319; doi:10.3390/microorganisms8071004)
Supplement: Supplementary file 1 [file microorganisms-08-01004-s001.zip › microorganisms-827284 suppl for XML conversion/Table S1.docx]

**Table S1**: Results of DESeq2 analysis for differentially abundant bacterial taxa between supragingival and salivary ecological niches (with supragingival samples as a reference).

| **Phylum** | **Genus** | **Species** | **log2FC** | **Adj. p value** |
| --- | --- | --- | --- | --- |
| Actinobacteria | *Actinomyces* | *aeruginosa* | 2.7363 | 5.017e-19 |
| Actinobacteria | *Actinomyces* | *dentalis* | 3.5755 | 5.286e-18 |
| Actinobacteria | *Actinomyces* | *dentalis_israelii_oricola_cluster** | 2.3707 | 2.785e-05 |
| Actinobacteria | *Actinomyces* | *graevenitzii* | -5.5117 | 1.308e-10 |
| Actinobacteria | *Actinomyces* | *israelii_massiliensis_gerencseriae* | 2.2690 | 4.878e-07 |
| Actinobacteria | *Actinomyces* | *odontolyticus_meyeri* | -1.7927 | 1.260e-08 |
| Actinobacteria | *Corynebacterium* | *matruchotii_mustelae* | 2.7648 | 3.394e-16 |
| Actinobacteria | *Rothia* | *aeria* | -0.8988 | 0.0292 |
| Actinobacteria | *Rothia* | *dentocariosa* | -1.3698 | 0.0001 |
| Actinobacteria | *Rothia* | *mucilaginosa_cluster* | -6.6148 | 9.245e-75 |
| Bacteroidetes | *Capnocytophaga* |  | 1.1186 | 0.0010 |
| Bacteroidetes | *Capnocytophaga* | *leadbetteri* | 0.7989 | 0.0256 |
| Bacteroidetes | *Capnocytophaga* | *ochracea* | 1.3529 | 0.0002 |
| Bacteroidetes | *Porphyromonas* |  | -1.0513 | 0.0090 |
| Bacteroidetes | *Porphyromonas* | *endodontalis* | -1.3519 | 0.0006 |
| Bacteroidetes | *Porphyromonas* | *pasteri** | -0.7434 | 0.0480 |
| Bacteroidetes | *Prevotella* | *aurantiaca* | -2.8775 | 1.064e-08 |
| Bacteroidetes | *Prevotella* | *intermedia** | 1.1980 | 0.0003 |
| Bacteroidetes | *Prevotella* | *loescheii* | 1.4643 | 0.0003 |
| Bacteroidetes | *Prevotella* | *melaninogenica* | -1.6714 | 8.299e-07 |
| Bacteroidetes | *Prevotella* | *nanceiensis* | -4.2637 | 4.281e-18 |
| Bacteroidetes | *Prevotella* | *oris* | -1.5652 | 8.031e-06 |
| Bacteroidetes | *Prevotella* | *oulorum* | -2.9380 | 4.713e-12 |
| Bacteroidetes | *Tannerella* | *forsythia* | 1.8259 | 3.871e-05 |
| Firmicutes | *[Clostridium]* | *cellobioparum_termitidis** | -4.7129 | 5.181e-21 |
| Firmicutes | *Abiotrophia* | *defectiva* | 0.7549 | 0.0133 |
| Firmicutes | *Bulleidia* | *extructa* | -2.6171 | 0.0007 |
| Firmicutes | *Bulleidia* | *moorei* | -1.5695 | 0.0001 |
| Firmicutes | *Catonella* | *morbi* | -1.6869 | 9.256e-08 |
| Firmicutes | *Dialister* |  | -1.2748 | 0.0001 |
| Firmicutes | *Dialister* | *invisus* | 0.6753 | 0.0476 |
| Firmicutes | *Enterococcus* | *saigonensis_hirae_olivae** | -0.8648 | 0.0002 |
| Firmicutes | *Granulicatella* | *adiacens_balaenopterae* | -0.8685 | 0.0006 |
| Firmicutes | *Lachnoanaerobaculum* | *orale** | 2.6047 | 3.579e-06 |
| Firmicutes | *Oribacterium* | *asaccharolyticum* | -6.5578 | 1.888e-16 |
| Firmicutes | *Oribacterium* | *sinus* | -5.5433 | 1.903e-31 |
| Firmicutes | *Parvimonas* |  | 1.3294 | 5.393e-05 |
| Firmicutes | *Peptococcus* |  | 1.1878 | 0.0005 |
| Firmicutes | *Ruminococcus* |  | -4.2904 | 5.158e-14 |
| Firmicutes | *Schwartzia* |  | -2.7215 | 4.911e-10 |
| Firmicutes | *Selenomonas* |  | -1.0448 | 0.0090 |
| Firmicutes | *Selenomonas* | *infelix* | -1.5077 | 4.114e-06 |
| Firmicutes | *Streptococcus* |  | 0.7616 | 0.0238 |
| Firmicutes | *Streptococcus* | *anginosus* | 1.5907 | 1.789e-07 |
| Firmicutes | *Streptococcus* | *thermophilus_vestibularis_salivarius** | -4.4051 | 6.488e-48 |
| Firmicutes | *Veillonella* |  | -1.1809 | 0.0098 |
| Firmicutes | *Veillonella* | *dispar* | -0.6428 | 0.0305 |
| Fusobacteria | *Fusobacterium* | *nucleatum* | 1.9086 | 1.158e-10 |
| Fusobacteria | *Fusobacterium* | *periodonticum** | 0.9492 | 0.0001 |
| Fusobacteria | *Leptotrichia* | *buccalis**_trevisanii** | 0.7926 | 0.0414 |
| Fusobacteria | *Leptotrichia* | *hofstadii* | 2.4717 | 3.0861e-10 |
| Fusobacteria | *Leptotrichia* | *wadei* | 2.4553 | 2.599e-10 |
| Proteobacteria | *Aggregatibacter* | *aphrophilussaccharolyticum** | 0.7522 | 0.0346 |
| Proteobacteria | *Aggregatibacter* | *pneumotrpica* | -5.5499 | 4.281e-18 |
| Proteobacteria | *Campylobacter* | *concisus_mucosalis_pinnipediorum** | -2.6732 | 5.0353e-17 |
| Proteobacteria | *Campylobacter* | *gracilis_showae_rectus* | -2.4167 | 4.261e-05 |
| Proteobacteria | *Cardiobacterium* | *hominis* | 2.1587 | 1.201e-10 |
| Proteobacteria | *Eikenella* | *corrodens* | 2.0488 | 9.122e-08 |
| Proteobacteria | *Haemophilus* | *influenzae** | -2.1580 | 1.733e-10 |
| Proteobacteria | *Haemophilus* | *parainfluenzae ** | -1.026 | 7.239e-05 |
| Proteobacteria | *Kingella* | *denitrificans** | 1.6626 | 3.579e-06 |
| Proteobacteria | *Lautropia* | *mirabilis** | -1.4879 | 1.034e-07 |
| Proteobacteria | *Neisseria* | *oralis* | -1.4087 | 0.0196 |
| Synergistetes | *TG5* |  | -1.2020 | 0.0098 |
| Tenericutes | *Acholeplasma* |  | -4.7222 | 2.042e-11 |
| Tenericutes | *Mycoplasma* |  | -2.2247 | 4.881e-09 |

OTUs merged at lowest taxonomic level. *Species annotation with lower than 97% identity using BLASTn to search the expanded Human Oral Microbiome Database (eHOMD) database. FC: fold change.
